# Human–Machine Collaboration in Diagnostics: Exploring the Synergy in Clinical Imaging with Artificial Intelligence

**DOI:** 10.3390/diagnostics13132162

**Published:** 2023-06-25

**Authors:** Antonia Pirrera, Daniele Giansanti

**Affiliations:** Centre TISP, ISS, 00166 Rome, Italy

Advancements in artificial intelligence (AI), thanks to IT developments during the COVID-19 pandemic, have revolutionized the field of diagnostics, particularly in clinical imaging [1,2,3]. Diagnostic imaging, whether it is applied *inside the human body for organs* [4] *or functional diagnostics* [5], in *tissues or within cells* [6,7], *or outside in the dermis*, is having fabulous developments thanks to AI [8]. With the advent of digital health and digital radiology (DR), digital pathology (DP), and digital dermatology (DD), healthcare professionals have gained powerful tools that enable faster, more accurate diagnoses. Increasingly innovative algorithms are developed in the medical imaging sector by researchers in basic data science research and implemented by IT specialists in increasingly innovative tools. These tools are increasingly used, and much is expected from them.

The DR, DP, and DD have evolved differently due to: (1) the different peculiarities of clinical diagnostics. (2) The different evolutions and developments of the digitization of the standardization of images (DR, for example, later than the DP [9,10,11,12]), which is the basis of the development and implementation of AI algorithms. (3) The different roles of the patient (for example, more operator-technologists in DD, more passive in DP and DR) [13,14].

DR has transformed [15] how medical images are captured, stored, and analyzed. It refers not only to traditional radiology but also to all the other fields of *organ and functional* imaging, ranging from echography to positron emission tomography. For example, traditional film-based X-rays in radiology and videotape recordings in echography have given way to digital imaging technologies, improving physicians’ workflow. Integrating AI algorithms with digital radiology has unlocked immense potential, empowering radiologists with intelligent systems that aid in detecting and interpreting abnormalities. Preliminary to this important development was the early development of the DICOM [11] standard, which facilitated the integration of these tools in every area and consequently made available a vast amount of image data for developing medical knowledge on AI.

Digital pathology [16] has emerged as a game-changer in histopathology and cytopathology, enabling the digitization and analysis of tissue and cell samples. By converting glass slides into digital slides and storing them into PACS (pathologists and cytologists) can interact easily through virtual scopes to discuss cases and for training.

However, this digitization has different characteristics in the two sectors of histology and cytology. The second one is more complex since the cytologist must use the focus function in the cytology, and its digital imitation requires an extension of the file. Compared to digital radiology, digital pathology has had greater inertia as regards standardization.

The specialized DICOM for digital pathology, DICOM Whole Slide Image (WSI) [12], has had a much longer release time and a more articulated adaptation of the manufacturers.

All this has meant that AI in digital pathology has certainly had a less rapid start.

DD has revolutionized the field of skin disease diagnosis and management [17,18,19]. Dermatologists now have access to powerful imaging technologies that capture high-resolution images of skin lesions and conditions. AI algorithms can assist in analyzing these images, identifying patterns, and suggesting potential diagnoses or treatment options. All this is also done thanks to mHealth mobile applications directly in the hands of the citizen integrated with the smartphone, who also becomes an *operator-technologist*. This aspect is new compared to DR and DP and opens a new paradigm in *Digital Health*.

Targeted searches on Pubmed give us an idea of the growth in the volume of studies, since the first applications of AI on the imaging at the date of this study.

Regarding the applications of AI in Pathology, the search with the key reported in *Box 1*, *position 1*, highlights 683 studies starting from 1989. Of these studies, 607 (*88.9%*) were carried out starting 1 January 2020. In all, there are 277 reviews (systematic and non-systematic).

Regarding the applications of AI in Dermatology, the search with the key reported in *Box 1*, *position 2*, highlights 97 studies starting from 2006. Of these studies, 83 (*85.6%*) were carried out starting 1 January 2020. In all, there are 42 reviews (systematic and not).

The DR includes, as explained, many sectors. To get an idea, we considered radiology and magnetic resonance.

Regarding the applications of AI in radiology imaging, the search with the key reported in *Box 1*, *position 3*, highlights 779 studies starting from 1983. Of these studies, 647 (*83.1%*) were carried out starting 1 January 2020. In all, there are 346 reviews (systematic and non-systematic).

Regarding the applications of AI in Magnetic Resonance imaging, the search with the key reported in *Box 1*, *position 4*, highlights 1132 studies starting from 1990. Of these studies, 1015 (89.7%) were carried out starting 1 January 2020. In all, there are 455 reviews (systematic and non-systematic).

This brief overview highlights how in these sectors: (1) scientific production and interest have accelerated during the COVID-19 pandemic. (2) The greatest production is in the DR sector. (3) There is a good percentage of review studies, indicating good progress in the stabilization process of topics of scientific interest (Figure 1).

Box 1 **The composite key used for the searches in Pubmed.**

*((pathology[Title/Abstract]) AND ((image[Title/Abstract]) OR (imaging[Title/Abstract]))) AND (Artificial Intelligence[Title/Abstract])*
*((dermatology[Title/Abstract]) AND ((image[Title/Abstract]) OR (imaging[Title/Abstract]))) AND (Artificial Intelligence[Title/Abstract])*
*((radiology[Title/Abstract]) AND ((image[Title/Abstract]) OR (imaging[Title/Abstract]))) AND (Artificial Intelligence[Title/Abstract])*
*((magnetic resonance[Title/Abstract]) AND ((image[Title/Abstract]) OR (imaging[Title/Abstract]))) AND (Artificial Intelligence[Title/Abstract])*

These developments can streamline the health domain processes and change the role and workflow of the professionals already involved in the decision-making process, such as radiologists, pathologists, cytologists, histologists, and dermatologists. However, other professional figures will also be able to make use of AI in the workflow, such as the medical radiology technician, the biological laboratory technician, and even the tattoo artists, who will be able to interact with other professional figures in diagnostics to monitor any problems [20] of this widespread practice [21].

However, a real integration has not yet been achieved in DR, DP, and DD, which implies acceptance of the actors, robust guidelines and stable regulations at an implementation and legislative level, well-defined workflows, adequacy for cyber security aspects, and ethical issues (the last two in some cases also interlaced), a better understanding of the AI algorithms of the actors though an explainable AI to have better control of the process and, more generally, consensus initiatives acting on multiple domains and involving all the actors and experts in the field [15,16,19]. There is a great need to discuss this area to exchange and share experiences with a 360-degree perspective, encapsulating opportunities, problems, and even failures. With this in mind, the Special Issue “Artificial Intelligence in Clinical Medical Imaging” [22] was launched.

## Conclusions

The COVID-19 pandemic has led to a terrifying acceleration in research and development on the application of AI. This applies to DP, DR, and DD.

However, creating ever more up-to-date tools based on increasingly performing innovative algorithms must be followed by initiatives in health domain integration that act on multiple domains. There is an increasing need for studies focused on AI in clinical imaging, also through synergistic initiatives, such as collections or Special Issues such as this one, which touch on successes and failures, opportunities, and bottlenecks.

## Figures and Tables

**Figure 1:**
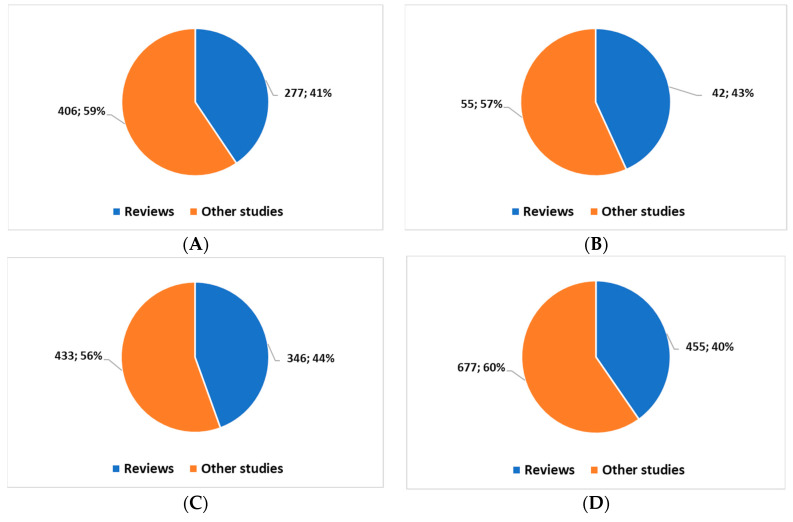
The volume of publications (at the date of this study) for the field of Pathology and AI (**A**); for the field of Dermatology and AI (**B**); for the field of radiology and AI (**C**); for the field of magnetic resonance and AI (**D**).

## Data Availability

Not applicable.
